# Proximal Proteomics Reveals Potential Regulators of Programmed Death-Ligand 1 in Human Cancer

**DOI:** 10.1021/acsomega.6c06622

**Published:** 2026-08-13

**Authors:** Fujin Shi, Danya Liu, Yaxiong Liu, Fan Li, Chris Zhiyi Zhang, Qiuli Li, Qiu-Hong Tian

**Affiliations:** † Department of Medical Oncology, The First Affiliated Hospital, 74653 Jiangxi Medical College, Nanchang University, Nanchang 330006, China; ‡ MOE Key Laboratory of Tumor Molecular Biology and State Key Laboratory of Bioactive Molecules and Druggability Assessment, Institute of Life and Health Engineering, College of Life Science and Technology, 47885 Jinan University, Guangzhou 510632, China; § Breast Disease Center, The First Affiliated Hospital, Jiangxi Medical College, Nanchang University, Nanchang 330006, China; ∥ Department of Head and Neck, Sun Yat-sen University Cancer Center, Guangzhou 510060, China

## Abstract

Programmed death-ligand 1 (PD-L1) is a key mediator of tumor immune evasion, but the protein networks governing its post-translational regulation remain incompletely understood. Here, we used PhastID-based proximity labeling to map the PD-L1 proximal proteome and integrated these data with pan-cancer proteomics and immunohistochemical analysis of clinical tumor samples. We found that C-terminal-proximal proteins were enriched in protein degradation, ER-associated quality control, and immune-related pathways, whereas N-terminal-proximal proteins were mainly associated with protein localization and intracellular transport. Integrated analysis identified CAND1, TXN, and FBXO2 as candidate PD-L1 regulators. Knockdown of CAND1, TXN, or FBXO2 reduced PD-L1 protein expression in cancer cells. Mechanistically, CAND1 interacted with PD-L1 and was detected in a complex containing PD-L1 and FBXO22. CAND1 depletion promoted K48-linked ubiquitination and degradation of PD-L1, while deletion of the PD-L1 intracellular domain or mutation of K271 and K281 attenuated PD-L1 ubiquitination. Functionally, CAND1 knockdown enhanced T cell-mediated tumor cell killing, and elevated CAND1 expression was associated with an immunosuppressive tumor microenvironment. Together, our findings define the PD-L1 proximal proteomic landscape and identify CAND1-mediated regulation of PD-L1 stability as a potential mechanism of tumor immune escape.

## Graphical abstract

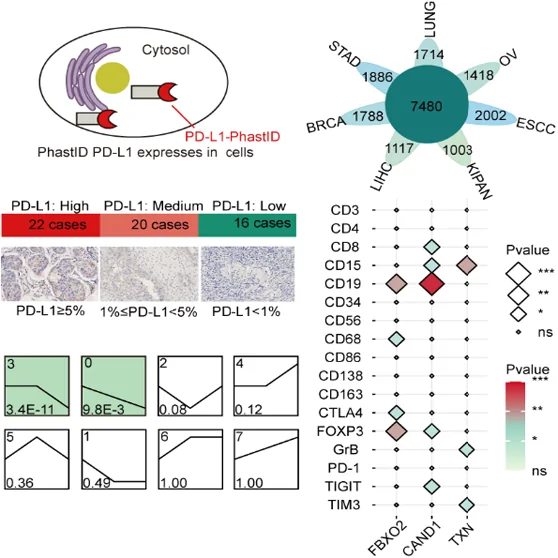

## Introduction

The PD-1/PD-L1 pathway has gained significant attention in the field of immunotherapy, showing unprecedented clinical responses in advanced cancers.^1^ In the tumor microenvironment (TME), cancer cells exhibit elevated PD-L1 expression in response to IFN-γ.^2^ Through its binding to PD-1, PD-L1 transduces inhibitory signals into T cells while delivering antiapoptotic signals to tumor cells, ultimately leading to T cell dysfunction and facilitating tumor cell survival.^3^ Anti-PD-1/PD-L1 therapy abrogates this immunosuppression and restores T cell-mediated antitumor responses. systematic analysis of PD-L1 expression and its protein–protein interaction network yields deeper mechanistic insights, which inform the rational design of combination strategies based on immune checkpoint blockade (ICB). Aberrant elevation of PD-L1 is linked to genomic amplification and translocation; additionally, transcription factors such as NF-κB, HIF-α, and STATs are dysregulated and aberrantly activated in the transcriptional regulation of PD-L1.^4^ Notably, posttranslational regulation of PD-L1 protein expression plays a critical role in driving its upregulation in cancer cells.^5^

Cullin Associated and Neddylation Dissociated 1 (CAND1) functions as a key assembly factor of the SCF (SKP1-CUL1-F-box protein) E3 ubiquitin ligase complex. It mediates the exchange of substrate-recognition F-box subunits within the SCF complex, thereby critically regulating the cellular repertoire and dynamics of SCF complexes.^6^ Emerging evidence has revealed that CAND1 mediates the deubiquitination of STAT3, thereby fostering immunosuppression and driving the progression of nonsmall cell lung cancer.^7^ Moreover, CAND1 has been shown to interact with GSTM3, which inhibits the ubiquitination and subsequent degradation of NRF2, consequently promoting the initiation and progression of gastric cancer.^8^ Consistent with its core molecular function, CAND1 participates in the regulation of protein turnover as an integral component of SCF (Skp1, Cullin, and F-box) E3 ubiquitin ligases.^9^ However, the roles of CAND1 in regulation of PD-L1 and modulation of TME have not been documented.

Proteomics-based network analysis is a systematic approach to constructing a data-driven framework for connecting biological pathways and disease traits. In this study, to identify the potential regulatory network of PD-L1, using pancancer tissue-based proteomics and PhastID^10^ to explore the proximal interactome of PD-L1. Eventually, we identified CAND1, TXN, and FBXO2 as potential regulators of PD-L1 expression and modulators for the immunosuppressive microenvironment.

## Materials and Methods

### Clinical Specimens

Clinical samples for proteomic analysis in this study were all obtained from patients admitted to Sun Yat-sen University Cancer Centre (SYSUCC, Guangzhou, China). Clinical tissue specimens were collected via surgical resection with written informed consent from all patients. For mass spectrometry analysis, the enrolled cases comprised the following cancer types: lung cancer (LUNG, *n* = 13), breast cancer (BRCA, *n* = 10), hepatocellular carcinoma (LIHC, *n* = 10), colorectal cancer (COAD/READ, *n* = 7), kidney cancer (KIPAN, *n* = 7), esophageal squamous cell carcinoma (ESCC, *n* = 4), gastric cancer (STAD, *n* = 4), and ovarian cancer (OV, *n* = 3).

### Protein Extraction and Sample Preparation for LC-MS/MS Analysis

To extract proteins from FFPE tissues after deparaffinization with xylene and rehydrating by concentration-gradient ethanol, the following procedures were used: adding high-concentration SDS lysate (0.3 mmol/L Tris-HCl, 4% SDS, pH 8.0) with preadded protease inhibitors, ultrasounding the sample at 40% amplitude for a total of 2 min, turn on for 2 s and turn off for 2 s, and centrifuging the sample at 12,000*g* for 10 min to remove insoluble debris. The supernatant was transferred to another clear tube and heated at 99 °C for 60 min to reverse the cross-linked protein. The extracted protein was precipitated using precooled acetone to remove impurities and was subjected to trypsin digestion using the FASP method.^11^ The precipitate was dissolved with the addition of 8 M urea. To reduce the disulfide bonds in the sample, DTT (Thermo Fisher, USA) was added to a final concentration of 50 mM and incubated at 37 °C for 1 h. Next, the sample was alkylated by adding iodoacetamide (Thermo Fisher, USA) to a final concentration of 150 mM and incubating at room temperature for 30 min in the dark. The samples were transferred to the rinsed FASP filters (Merck, Germany) and centrifuged at 12,000*g* for 15 min. All subsequent centrifugation steps were performed under the same conditions. The FASP filters were then washed and centrifuged multiple times with 200 μL of 8 M urea and 50 Mm TEAB (Thermo Fisher, USA), respectively. The sample was treated with trypsin and allowed to digest overnight at 37 °C with shaking at 1200 rpm. Following overnight digestion, the peptides were freeze-dried and reconstituted with 0.5% TFA (Thermo Fisher, USA). The samples were desalted using C18 Spin Columns, following the manual protocol. After desalting, the samples were freeze-dried and reconstituted with 0.1% FA (Thermo Fisher, USA). An equal mass of peptides was added to the iRT instrument (Biognosys, Switzerland) and transferred to the sample tube for mass spectrometry.

### Protein Extraction and Sample Preparation for AP-MS Analysis

Cells were harvested and lysed on ice for 15 min, followed by centrifugation at 12,000*g* for 10 min at 4 °C to remove insoluble debris. The clarified lysates were incubated with streptavidin magnetic beads (Thermo Fisher, USA) at 4 °C for 2 h with gentle rotation to enrich biotinylated proteins. The beads were then washed sequentially with 2% SDS, TBST, and TBS to remove nonspecifically bound proteins.

For on-bead digestion, the beads were resuspended in 50 mM TEAB, reduced with 20 mM DTT at 37 °C for 60 min, and alkylated with 40 mM IAA at 25 °C for 30 min in the dark. After washing three times with 50 mM TEAB, the beads were resuspended in 50 mM TEAB and digested with trypsin at 37 °C for 16 h with shaking. The resulting peptides were desalted using C18 Spin Columns according to the manufacturer’s instructions, freeze-dried, and reconstituted in 0.1% formic acid for LC-MS/MS analysis.

All raw MS data were searched with MaxQuant against the human proteome (Uniprot canonical protein sequences downloaded May 29, 2024) Peptides, proteins, and PTMs were filtered to 1% false discovery rate in MaxQuant (V1.6.5.0). MaxQuant default parameters were used with the exception that label-free quantification was turned on with match between runs set to 1 min, and for antibiotin purified samples a variable mass addition of biotin, and uses the iBAQ value as the quantitative basis. For data processing, delete the five common cytoplasmic contaminant proteins “ACACA_HUMAN,” “ACACB_HUMAN,” “MCCA_HUMAN,” “PCCA_HUMAN,” and “PYC_HUMAN”. To calculate the FOT values for each of the replicates based on iBAQ, we calculate the total iBAQ for each replicate: for each replicate,calculate the sum of all iBAQ values. Calculate the FOT values: for each protein, in each replicate, divide the iBAQ value of the protein by the total iBAQ of the corresponding replicate.

The formula can be expressed as

$$\mathrm{FO}T_{n∼i}=\frac{\mathrm{iBA}Q_{n∼i}}{\sum \mathrm{iBA}Q_{n}}$$

To classify proteins as C-terminal or N-terminal preferential interactors, a protein must meet one of the following two criteria: (1) identified in over 50% of replicates in the compared groups; (2) fold change >2 compared to the control group’s FOT value.

### Mass Spectrometry

Tryptic peptides were reconstituted in 0.1% formic acid and analyzed on an Orbitrap Fusion Lumos mass spectrometer (Thermo Fisher Scientific) coupled to a nanoflow liquid chromatography system. Peptides were separated at a flow rate of 300 nL/min using a 60 min gradient. The mass spectrometer was operated in data-dependent acquisition (DDA) mode. Full MS scans were acquired in the Orbitrap over an *m*/*z* range of 350–1500 at a resolution of 120,000. Precursor ions were isolated in the quadrupole with an isolation window of 1.6 *m*/*z* and fragmented by higher-energy collisional dissociation (HCD) using a normalized collision energy of 32%. MS/MS spectra were acquired in the Orbitrap at a resolution of 15,000, with a maximum injection time of 230 ms. Raw LC-MS/MS data were processed using MaxQuant software. Spectra were searched against the UniProt human proteome database. Trypsin was specified as the digestion enzyme, with up to two missed cleavages allowed.

### Series Test of Cluster

Gene expression pattern analysis was performed by Short Time-series Expression Miner software­(STEM)^12^ on the OmicShare tools platform. The parameters were set as follows: (1) Maximum Unit Change in model profiles between time points is 1; (2) Maximum output profiles number is 8­(similar profiles will be merged); (3) Minimum ratio of fold change of DEGs is no less than 2.0. The first time point was used as the control to calculate the fold change (FC) of all samples. After this, the expression level of the first sample will be 0. Samples with values greater than 0 are considered to have upregulated expression, while samples with values less than 0 are considered to have downregulated expression. Trends with a *P*-value <0.05 are considered significant.

### Mfuzz Clustering Analysis

Fuzzy c-means clustering was further performed using the Mfuzz package in R to identify protein expression patterns associated with PD-L1 expression levels. Protein abundance data were first filtered to remove proteins with excessive missing values, and the remaining missing values were imputed before clustering analysis. The expression matrix was then standardized using the standardize function in Mfuzz to ensure comparable expression dynamics across proteins. The fuzzification parameter was estimated using the mestimate function, and the optimal number of clusters was determined according to cluster stability and expression pattern separation. Fuzzy c-means clustering was performed using the mfuzz function. Proteins were assigned to the cluster with the highest membership score, and proteins with high membership values were considered representative members of each cluster. Clustered expression patterns were visualized using the mfuzz plot function. Clusters significantly associated with PD-L1 expression levels were selected for subsequent functional enrichment and intersection analyses.

### Pancaner Proteomic Analysis

The online tools Sangerbox,^13^ and BioLadder tool^14^ were used to evaluate the differences in RNA and protein levels between tumor and normal tissues, as well as their correlation with clinical status. The R packages survminer, Mfuzz and survivoR were used to plot Kaplan–Meier survival curves. Statistical significance was determined using the log-rank test.

### Cell Lines

The human CRC cell line RKO was cultured in RPMI-1640 (Gibco, USA), while the human ccRCC cell line 786-O, the 293T cell line, and the human ESCC cell line KYSE-150 was cultured in DMEM (Gibco, USA). All culture media were supplemented with 10% FBS (Gibco, USA) and 1% penicillin-streptomycin (Gibco, USA). Cells were incubated at 37 °C in 5% CO_2_. After transfection with Lipofectamine 3000 for 6 h, cells were cultured for an additional 42 h, followed by the addition of 100 μM biotin for 6 h.

### RNA Extraction and qPCR

Total RNA was extracted from cells using the Trizol reagent (Invitrogen, USA) following the manufacturer’s instructions. The RNA was then converted to cDNA using a cDNA synthesis kit (Yeasen, China) and subjected to qPCR experiments with the SYBR Green mix (Yeasen, China) with a Bio-Rad Real-Time detection system (Bio-Rad, USA). The relative expression levels of target genes were calculated and normalized using the 2^–ΔΔ*Ct*^ method. GAPDH was used as a reference gene. The primer sequences used are as follows: CD274-F: TGGCATTTGCTGAACGCATTT; CD274-R: TGCAGCCAGGTCTAATTGTTTT; GAPDH-F: GTCTCCTCTGACTTCAACAG-CG; GAPDH-R: ACCACCCTGTTGCTGTAGCCAA; TXN-F: GTGAAGCAGATC-GAGAGCAAG; TXN-R: CGTGGCTGAGAAGTCAACTACTA; CAND1-F: TGAC- CTGTCTACTTCCCCAGT; CAND1-R: CCACAGCTCATAACCAGATGG.

### Western Blotting

For Western blot analysis, cells were collected and washed with PBS, then lysed in RIPA buffer (Cell Signaling Technology, USA). After incubation on ice for 30 min, the lysates were centrifuged at 12,000*g* for 10 min at 4 °C. The protein solution was quantified using a BCA Protein Assay Kit (Thermo Fisher, USA). Protein samples were added with 2× loading buffer and heated at 100 °C for 10 min, separated with SDS-polyacrylamide gel electrophoresis (SDS-PAGE), transferred to a PVDF membrane, and blocked with 5% nonfat milk in Phosphate Buffered Saline with 0.1% Tween-20 (PBST) for 1 h at room temperature. The membranes were probed with the corresponding primary antibodies at 4 °C overnight, including FBXO2 primary antibody (Proteintech, China), PD-L1 primary antibody (Cell Signaling Technology, USA), CAND1 primary antibody (Cell Signaling Technology, USA), TXN primary antibody (Cell Signaling Technology, USA), and GAPDH (Proteintech, China). Following extensive washing, the membrane was incubated with HRP-labeled secondary antibody for 2 h at room temperature. Target protein bands were visualized with an ECL chemiluminescence kit (Bio-Rad, USA) and imaged with a Luminescence Imaging System (Tanon, China).

### Enrichment and Network Analyses

Gene Ontology (GO) term enrichment analysis was performed using ClueGO v2.5.4 in Cytoscape v3.7.1 platform. Enrichment and network analyses were conducted across the three principal GO domains: molecular function (MF), biological process (BP), and cellular component (CC).

### Co-Immunoprecipitation

For coimmunoprecipitation (Co-IP) assays between PD-L1 and CAND1, cells were lysed in RIPA buffer (1% Triton X-100, 100 mM Tris-HCl pH 8.8, 100 mM NaCl, 0.5 mM EDTA) freshly supplemented with a complete protease inhibitor cocktail (Bimake). Cell lysates were incubated with the indicated antibodies at 4 °C for 4 h, followed by the addition of Anti-HA Nanobody Magarose Beads (AlpalifeBio, China) and further incubation at 4 °C for 2 h. Beads were washed three times with Dilution/Wash buffer (50 mM Tris-HCl pH 7.5, 150 mM NaCl, 1 mM EDTA) and then analyzed by Western blotting.

### T Cell-Mediated Cytotoxicity Assay

To evaluate the effect of CAND1 on tumor cell susceptibility to T cell-mediated killing, a CFSE/PI-based cytotoxicity assay was performed. Briefly, tumor cells were harvested and labeled with CFSE following the manufacturer’s instructions, then washed thoroughly to remove unbound dye. CFSE-labeled tumor cells were seeded into culture plates and cocultured with T cells at an effector-to-target ratio of 5:1 for 24 h. After coculture, cells were harvested and stained with propidium iodide (PI) to evaluate tumor cell death. Flow cytometry analysis was conducted to quantify the percentage of PI-positive cells among the CFSE-positive tumor cell population. T cell-mediated cytotoxicity was calculated as the proportion of CFSE^+^PI^+^ tumor cells.

### Immunohistochemistry (IHC)

Tissue microarray (TMA) slides underwent deparaffinization with xylene and subsequent rehydration. Antigen retrieval was performed with EDTA buffer (pH 9.0) and boiled in microwave for 2.5 min. Endogenous peroxidase activity was quenched with 3% hydrogen peroxide and was blocked by blocking buffer. Primary antibodies were then applied, and the sections were incubated overnight at 4 °C. After three rounds of washing with PBST buffer, the tissue sections were incubated with anti-Rabbit or mouse HRP secondary antibody (Zhongshan Golden Bridge Biotechnology, China) for 20 min. Markers were stained with DAB and staining intensity observed under a microscope. Hematoxylin was used to stain the nuclei. HALO analysis software (PANOVUE, China) was used for quantification.

### Statistical Analysis

Numerical data are presented as means ± SEM; all data analyses were performed using GraphPad Prism (version 7.0, GraphPad Software, Inc.). One-way analysis of variance was used to analyze the statistical differences among the groups with P-values indicated in the related graphs. The level of statistical significance was set at a *p* < 0.05. All assays were carried out at least three independent times with the same results.

## Result

### Labeling of the PD-L1 Interactome in 293T Cells

To explore the PD-L1 interactome in 293T cells, we employed a novel BioID system termed Pyrococcus horikoshii BPL (PhBPL)-Assisted Biotin Identification (PhastID).^10^ To identify the cellular interacting partners of PD-L1, we constructed a PD-L1-PhastID-HA fusion plasmid, transiently transfected it into 293T cells and HepG2 cells, and labeled proximal proteins with 100 μM biotin for 6 h. The PD-L1 proximal proteome was subsequently identified via mass spectrometry (Figure 1A, Supporting Tables 1–4). To capture proteins specifically interacting with the N-terminal or C-terminal region of PD-L1, we generated N- or C-tagged PhastID-PD-L1 constructs and used PhastID alone as a negative control (Figure 1B). Confocal analyses confirmed that PhastID-PD-L1 fusion did not alter the subcellular localization of PD-L1 (Figure 1C). Western blot analysisBLE showed comparable levels of biotinylated proteins between N-PhastID-PD-L1 and C-PhastID-PD-L1 groups (Figure 1D, Figure S1A–S1B).

**Figure 1:**
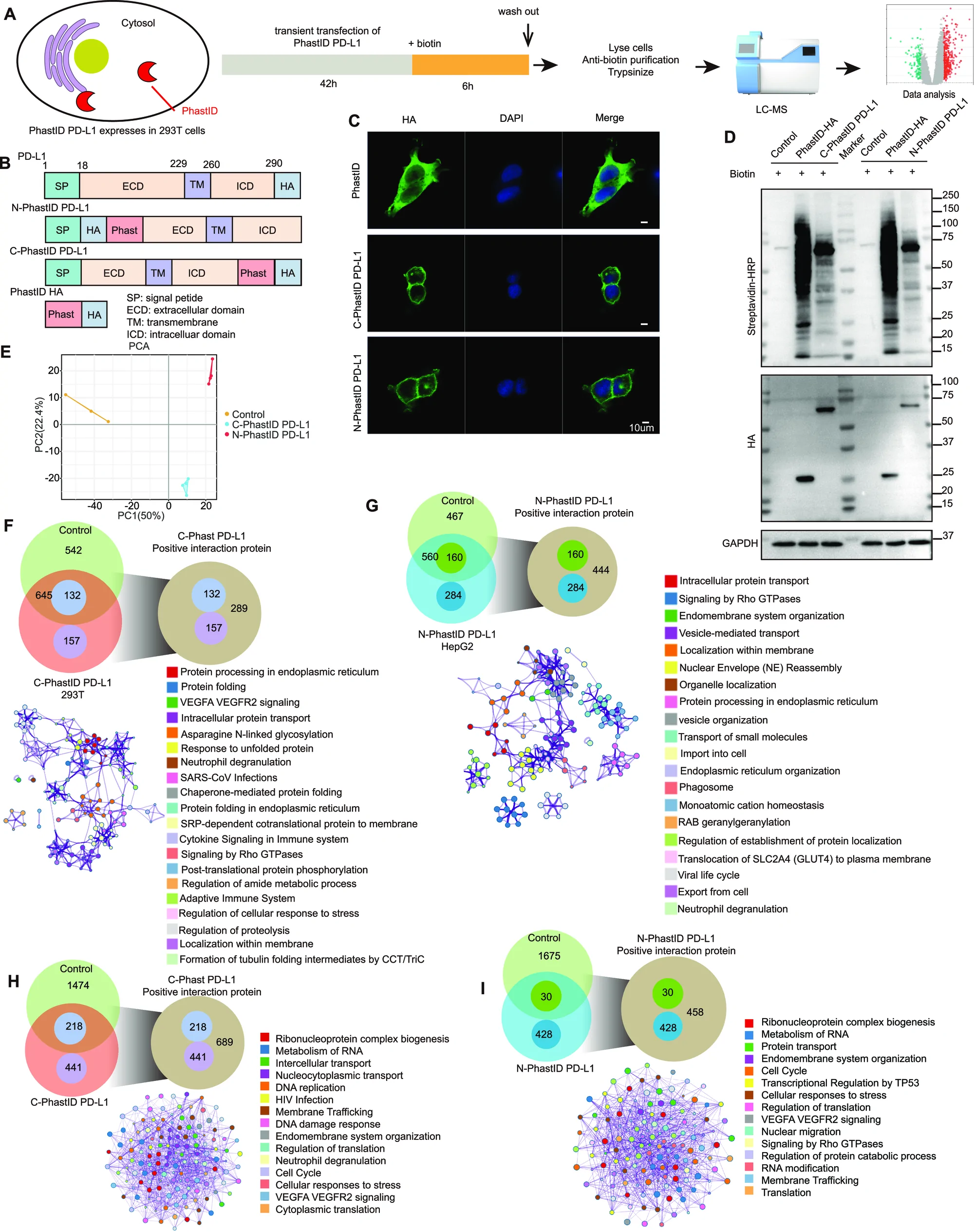
PD-L1-PhastID-mediated proximity-dependent biotinylation in human 293T cells. (A) Schematic illustration of the proximity-dependent biotinylation workflow for identifying PD-L1-associated proteins. 293T cells expressing PD-L1-PhastID were incubated with biotin, followed by cell lysis, streptavidin-based enrichment of biotinylated proteins, on-bead trypsin digestion, and liquid chromatography-tandem mass spectrometry (LC-MS/MS) analysis. (B) Schematic representation of PD-L1-PhastID fusion constructs, including N-terminal and C-terminal PhastID tagging strategies. (C) Confocal microscopy images showing the subcellular localization of PD-L1-PhastID fusion proteins in 293T cells. (D) Western blot analysis confirming the expression of N-terminal and C-terminal PhastID-PD-L1 fusion proteins, as well as the biotinylation of proximal proteins in 293T cells. Streptavidin-HRP was used to detect biotinylated proteins. (E) Principal component analysis (PCA) distribution of mass spectrometry data. (F, G) Venn diagrams of biotinylated proteins detected in 293T cells expressing C-PhastID-PD-L1 (F) and N-PhastID-PD-L1 (G). Proteins with a fold change >2 in the intersection were considered positive, and specifically expressed proteins were directly designated as positive. (H, I) Venn diagrams of biotinylated proteins detected in HepG2 cells expressing C-PhastID-PD-L1 (H) and N-PhastID-PD-L1 (I). Meta-panorama-based gene ontology (GO) enrichment analysis (ColorByCluster) was presented.

Biotinylated proteins were identified by mass spectrometry, and principal component analysis (PCA) revealed clear separation between the experimental groups (Figure 1E). A total of 289 specific proteins were identified at the C-terminal of PD-L1 (Figure 1F), whereas 444 specific proteins were detected at the N-terminal (Figure 1G). Metascape (https://metascape.org) analyses demonstrated that both N- and C-terminal associated proteins were involved in endoplasmic reticulum (ER)-related pathways. Furthermore, proteins interacting with C-terminal of PD-L1 were specifically enriched Asparagine glycosylation and adaptive immune system, whereas those interacting with N-terminal of PD-L1 were involved in Rho GTPases and nuclear envelope (NE) reassembly.

To further characterize the PD-L1 proximal proteome in tumor cells, we performed parallel PhastID-based proximity labeling in the HepG2 hepatocellular carcinoma cells. Mass spectrometry analysis uncovered a comprehensive repertoire of PD-L1-proximal proteins in HepG2 cells. Compared with the PhastID control group, 689 proteins were specifically enriched in the C-PhastID-PD-L1 group and 458 proteins were uniquely enriched in the N-PhastID-PD-L1 group. Pathway enrichment analysis revealed that C-terminal-proximal proteins in HepG2 cells are primarily implicated in ribonucleoprotein complex biogenesis, RNA metabolism, nucleocytoplasmic transport, DNA replication, membrane trafficking, DNA damage response, and cellular stress responses (Figure 1H). In comparison, N-terminal-proximal proteins are associated with ribonucleoprotein complex biogenesis, RNA metabolism, protein transport, endomembrane system organization, cell cycle regulation, TP53-dependent transcriptional regulation, translation, and Rho GTPase-mediated signaling (Figure 1I).

### Localization and Functional Divergence of PD-L1-Interacting Proteins

To further delineate the functional differences between the N- and C-terminal domains of PD-L1, we compared the interactomes identified by N- and C-PhastID-PD-L1. Among the 611 putative PD-L1-associated proteins, 122 were commonly enriched in both tagging strategies (Figure 2A), indicating a shared core interactome. Protein–protein interaction (PPI) network analysis further characterized these PD-L1-associated proteins (Figure 2B), many of which are implicated in PD-L1 regulation and tumor progression. Notably, several previously reported PD-L1 regulatory proteins, including CDK1, STT3A/STT3B, mTOR, and CD99, were identified within this network, validating the biological relevance and reliability of PhastID-based interactome mapping.

**Figure 2:**
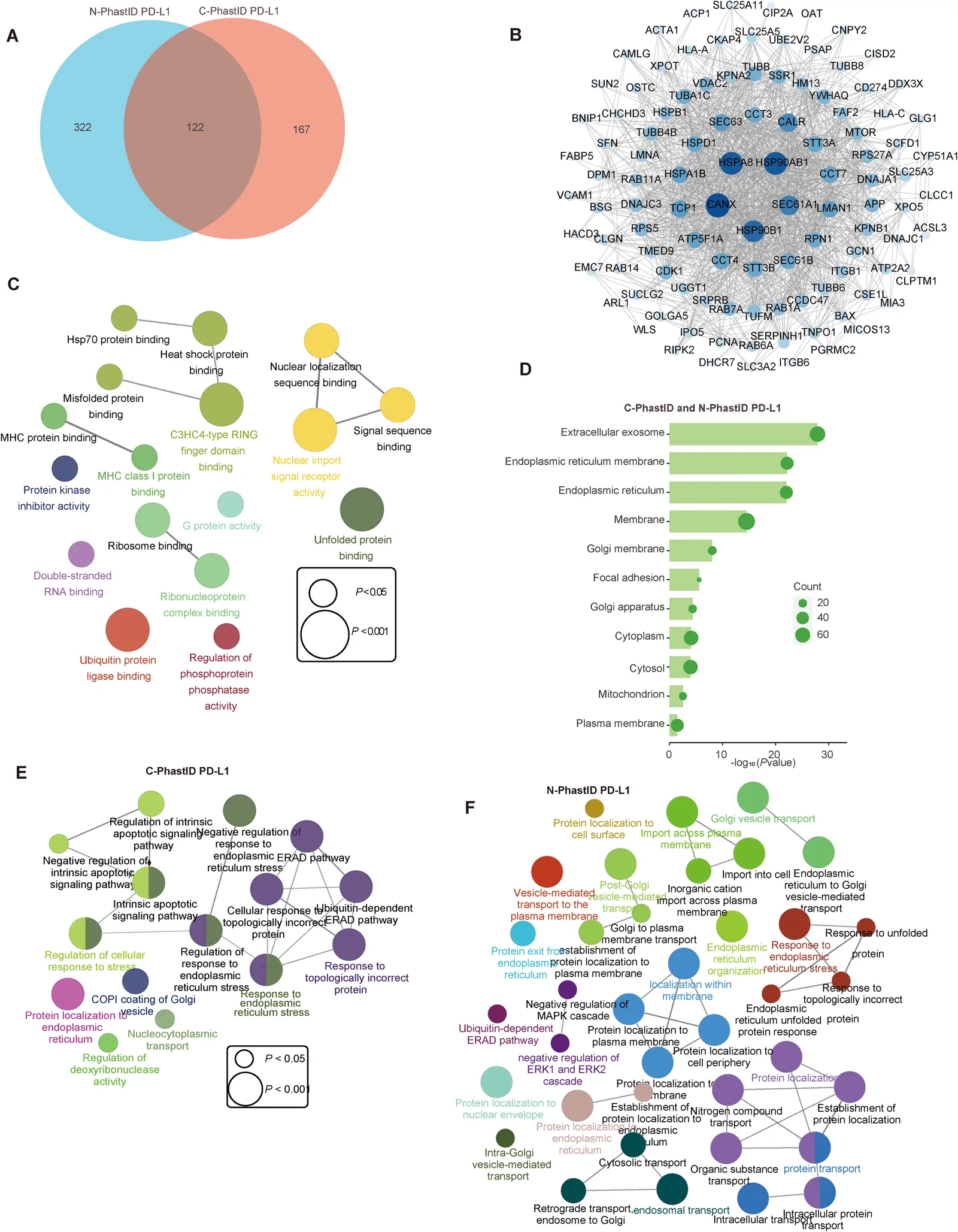
Subcellular and domain-specific PD-L1 interactome in living human cells identified by PhastID-mediated biotinylation. (A) Venn diagram comparing biotinylated proteins identified in 293T cells expressing N-terminally tagged PhastID-PD-L1 and C-terminally tagged PhastID-PD-L1, illustrating shared and domain-specific PD-L1-associated proteins. (B) Protein–protein interaction (PPI) analysis of proteins coenriched in both N-PhastID-PD-L1 and C-PhastID-PD-L1 groups. (C) ClueGO-based molecular function enrichment analysis of proteins biotinylated by both N-PhastID-PD-L1 and C-PhastID-PD-L1. Node colors indicate functionally grouped networks based on shared GO terms. (D) GO cellular component enrichment analysis of proteins commonly biotinylated by N-PhastID-PD-L1 and C-PhastID-PD-L1, showing the major subcellular localizations associated with PD-L1. (E-F) ClueGO molecular function pathway enrichment analyses of proteins specifically biotinylated by N-PhastID-PD-L1 (E) and C-PhastID-PD-L1 (F).

Gene Ontology (GO) enrichment analysis showed that these proteins are involved in pathways related to heat shock protein binding, MHC protein binding, and various enzyme activity-associated biological processes (Figure 2C). Cellular component classification of biotinylated proteins mainly included extracellular exosomes and the endoplasmic reticulum (Figure 2D), which is consistent with the subcellular localization of PD-L1. Notably, 80% of the interactions in N-PhastID or C-PhastID-PD-L1-expressing cells were uniquely detected (Figure 2B). Enrichment analyses of the distinct N- and C-terminal PD-L1-associated proteins uncovered the regulatory network of PD-L1. Specifically, C-terminal-associated pathways primarily mediate PD-L1 targeting to the endoplasmic reticulum for degradation via the ERAD pathway, while N-terminal-associated functions are significantly enriched in protein localization and transport processes (Figure 2E–F).

To determine whether the PD-L1-associated proteomic network is conserved in tumor cells, we further analyzed the PD-L1 proximal proteome profiled in HepG2 cells. N- and C-terminal PhastID-PD-L1 labeling identified both overlapping and region-specific PD-L1-interacting proteins (Figure S1C). Protein–protein interaction (PPI) network analysis of these shared proteins constructed a tightly connected interaction network (Figure S1D). Gene Ontology (GO) molecular function enrichment revealed prominent enrichment in tRNA binding, translation regulator activity, nucleic acid binding, ribosome binding, ribonucleoprotein complex binding, and other RNA-binding-associated functions (Figure S1E). Cellular component annotation further showed that the overlapping proteins predominantly localize to the cytoplasm, cytosol, nucleus, nucleoplasm, mitochondria, extracellular exosomes, Golgi apparatus, and endoplasmic reticulum (Figure S1F).

Additional GO biological process analysis demonstrated that proteins captured by C-terminal PhastID-PD-L1 are enriched in cellular amide metabolism, ER-to-Golgi vesicle-mediated transport, ncRNA processing, tRNA aminoacylation, and ribonucleoprotein complex and ribosome biogenesis (Figure S1G). In contrast, N-terminal-enriched proteins are primarily involved in ubiquitin-dependent protein catabolism, mitochondrial respiratory chain complex assembly, and chaperone-mediated protein folding (Figure S1H). Collectively, these results indicate that the PD-L1 proximal proteome in HepG2 cells is closely associated with RNA metabolism, protein trafficking, and mitochondrial regulatory processes.

### Pancancer Proteomics Combined with PhastID Proximity Labeling Reveals the PD-L1 Interaction Network

To precisely identify potential PD-L1-interacting proteins in clinical samples, we integrated PD-L1-associated proteomic data with a pancancer proteomic data.^15^ This cohort included 58 patients with solid tumors representing eight distinct cancer types (Figure 3A). Label-free proteomic analysis of tumor cell-enriched formalin-fixed, paraffin-embedded (FFPE) tissue sections was performed using the same mass spectrometer across all samples. A total of 10,816 proteins were detected across all samples, with each sample containing 7655 to 9153 proteins (Figure 3B, Supporting Table 5). Notably, the number of proteins detected in kidney and liver cancer tissues was lower than the overall average, a finding consistent with PCA and clustering analysis results (Figure 3C–D). To reduce bias caused by cancer heterogeneity, we used the intersection of proteins across all tumor types as the basis for subsequent analyses. The number of proteins detected in each tumor type ranged from 8483 to 9482 (Figure 3E). A total of 7480 proteins were commonly detected across all cancer types, and consistent quality control across different tumors also revealed unique protein expression profiles in certain cancer types (Figure 3F–G).

**Figure 3:**
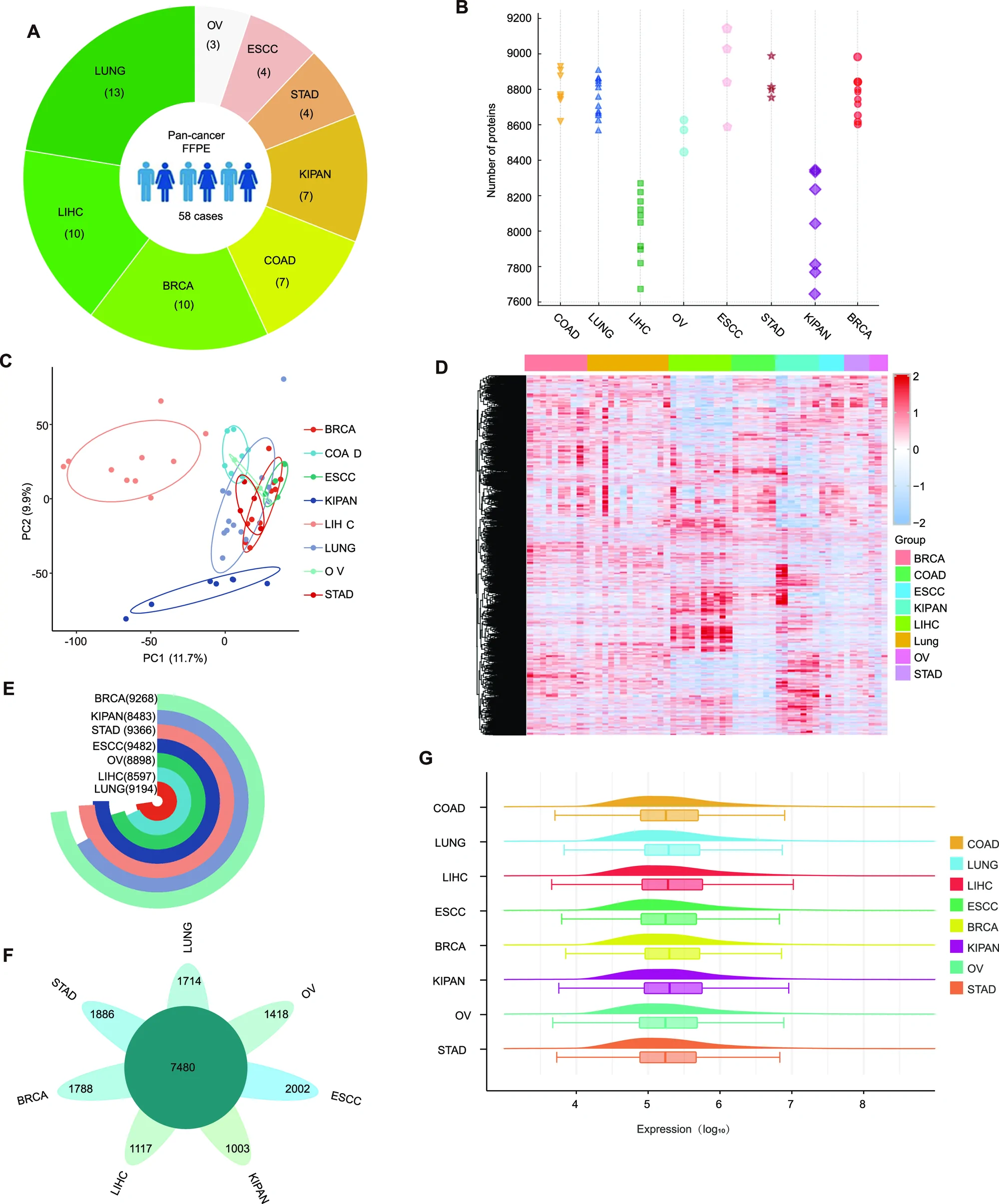
Proteomic landscape of pan-cancer tumor tissues. (A) Distribution of tumor samples across eight cancer types in the pan-cancer cohort (total *n* = 58). (B) Number of proteins identified by LC-MS/MS in each sample, ranging from 7655 to 9153. (C) PCA showing the global proteomic profiles of 58 solid tumor samples across eight cancer types. (D) Heatmap illustrating hierarchical clustering of protein expression patterns across different tumor types based on normalized proteomic data. (E) Number of proteins identified in each tumor type after excluding low-confidence proteins. (F) Venn diagram showing the overlap of proteins detected across all cancer types, highlighting a core set of 7480 commonly identified proteins and cancer type-specific proteins. (G) Distribution of expression levels of the 7480 commonly detected proteins across different cancer types, indicating overall stability of global protein expression patterns among tumors.

In addition, PD-L1 expression was evaluated by immunohistochemistry (IHC) in tumor tissues from the 58 patients (Figure 4A–B, Supporting Table 6). Based on PD-L1 staining levels, patients were stratified into three groups: low-expression (PD-L1 < 1%, *n* = 16), medium-expression (1% ≤ PD-L1 < 5%, *n* = 20), and high-expression (PD-L1 ≥ 5%, *n* = 22). Statistical analysis confirmed significant differences in PD-L1 expression among these groups (Figure 4C), validating this stratification strategy.

**Figure 4:**
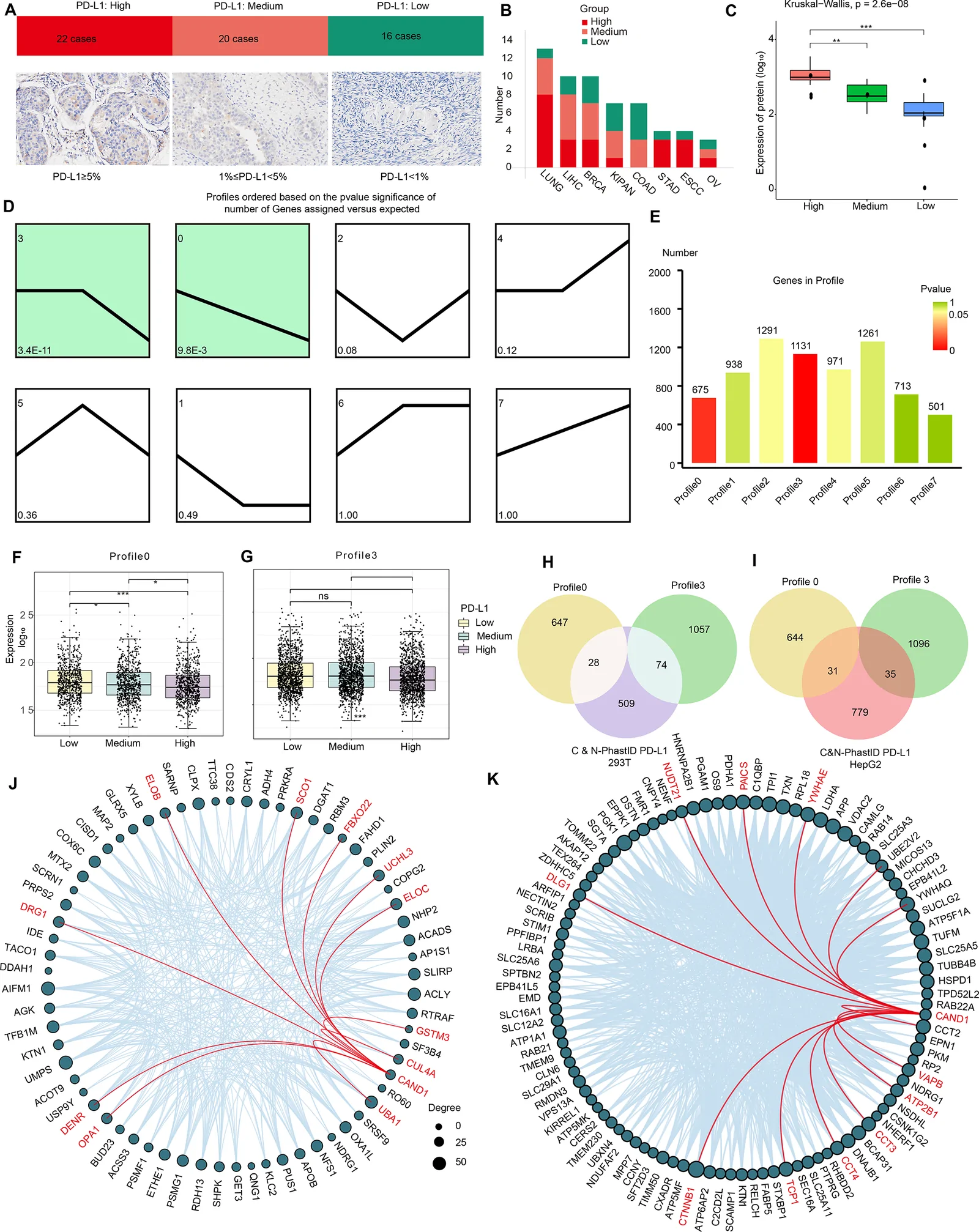
Integration of pan-cancer proteomics and PhastID proximity labeling reveals the PD-L1 interaction network. (A) Representative IHC staining images of PD-L1 in tumor tissues from 58 patients, stratified into high-, medium-, and low-expression groups based on PD-L1 IHC scores. (B) Distribution of tumor samples across PD-L1 high-, medium-, and low-expression groups. (C) Comparison of PD-L1 expression levels among the three groups, analyzed by Kruskal–Wallis test. (D) Trend analysis of 7480 proteins using the Series Test of Cluster algorithm, revealing eight distinct expression patterns across PD-L1 low, medium, and high groups. Each profile represents a unique expression trend, with colors indicating statistically significant trends. (E) Number of proteins in each of the eight modules; colors represent significant trends. (F–G) Analysis of protein expression level differences in Module 0 and Module 3, stratified by PD-L1 expression levels. (H) Venn diagram showing the overlap between proteins from PD-L1-associated expression profiles (Modules 0 and 3) and PD-L1 proximal proteins identified by PhastID-mediated biotinylation in 293T cells. (I)­Venn diagram showing the overlap between proteins from PD-L1-associated expression profiles (Modules 0 and 3) and PD-L1 proximal proteins identified by PhastID-mediated biotinylation in HepG2 cells. (J) PPI network of 66 genes identified by the overlap between pan-cancer proteomics and biotin proximity labeling in HepG2 cells. (K) PPI network of 101 genes identified by the overlap between pan-cancer proteomics and biotin proximity labeling in 293T cells.

To identify proteins associated with PD-L1 expression in clinical tumor samples, 7480 commonly quantified proteins were subjected to trend analysis according to PD-L1 expression levels. Proteins with similar expression patterns were clustered into eight modules (Figure 4D). Among these modules, profiles 0 and 3 showed the strongest correlation with PD-L1 expression, comprising 675 and 1131 proteins, respectively, whereas the remaining modules showed weaker or no significant association (Figure 4E). Further comparison of protein abundance across PD-L1-low, -medium, and -high groups confirmed that proteins in profiles 0 and 3 displayed significant PD-L1-related expression trends (Figure 4F–G).

To address the potential influence of tumor heterogeneity on pan-cancer proteomic analysis, we further conducted a cancer type-specific validation analysis using an independent ESCC proteomic data set.^16^ In this external cohort, quantified proteins were stratified according to PD-L1 expression levels and subjected to trend clustering analysis (Supporting Tables 7–8). This analysis identified six expression clusters with distinct PD-L1-associated patterns (Figure S2A). Functional enrichment analysis showed that these ESCC-specific clusters were mainly involved in protein transport, mRNA processing, proteasome-mediated ubiquitin-dependent protein catabolic process, cell migration, DNA-templated transcriptional regulation, cell division, intracellular signal transduction, rRNA processing, DNA damage response, apoptosis, inflammatory response, protein ubiquitination, and protein stabilization (Figure S2B). Notably, CAND1 showed a positive association with PD-L1 expression in ESCC samples and was linked to ubiquitin-dependent protein catabolism-related processes. These results indicate that the PD-L1-associated proteomic network identified in the pan-cancer cohort is not solely driven by intertumoral heterogeneity, but is at least partially preserved within a single tumor type.

To obtain high-confidence PD-L1-associated proteins with both clinical relevance and spatial proximity, we intersected the proteins from profiles 0 and 3 with the PD-L1 proximal proteomes identified by PhastID labeling. Intersection with the 293T C- and N-PhastID-PD-L1 data set identified 101 overlapping proteins (Figure 4H), whereas intersection with the HepG2 C- and N-PhastID-PD-L1 data set identified 66 overlapping proteins (Figure 4I). PPI network analysis further revealed distinct PD-L1-associated interaction networks in HepG2 and 293T cells (Figure 4J–K). Notably, comparison of the overlapping candidates from both cell lines identified three shared proteins, among which CAND1 was correlated with PD-L1 in clinical tumor samples (Figure S2C).

### Clinical Significance of CAND1 and TXN Expression in Human Cancers

To evaluate the clinical relevance of CAND1 and TXN, we performed differential expression analysis and Kaplan–Meier survival analysis using The Cancer Genome Atlas (TCGA) data sets. The data showed that CAND1 was significantly overexpressed in tumor tissues compared with normal tissues in lung cancer, breast invasive carcinoma (BRCA), colon adenocarcinoma/rectum adenocarcinoma (COAD/READ), head and neck squamous cell carcinoma (HNSC), pan-kidney cohort (KICH, KIRC, and KIRP; KIPAN), liver hepatocellular carcinoma (LIHC), and esophageal carcinoma (ESCA) (Figure 5A). To further explore the relationship between CAND1 and PD-L1, correlation analysis was performed using the TIMER2.0 platform. CAND1 expression was significantly positively correlated with PD-L1 mRNA levels across multiple cancer types (Figure 5B), consistent with their association identified in our proteomic analyses. Kaplan–Meier survival analyses demonstrated that high CAND1 expression was significantly associated with poorer overall survival in patients with BRCA, LIHC, and lung cancer (Figure 5C–E). On the other hand, TXN mRNA expression was significantly dysregulated between tumor and normal tissues in several cancer types (Figure 5F). Correlation analysis revealed a significant association between TXN and PD-L1 expression across multiple TCGA cohorts (Figure 5G). Importantly, elevated TXN expression was significantly correlated with unfavorable prognosis in patients with BRCA, LIHC, and lung cancer (Figure 5H–J).

**Figure 5:**
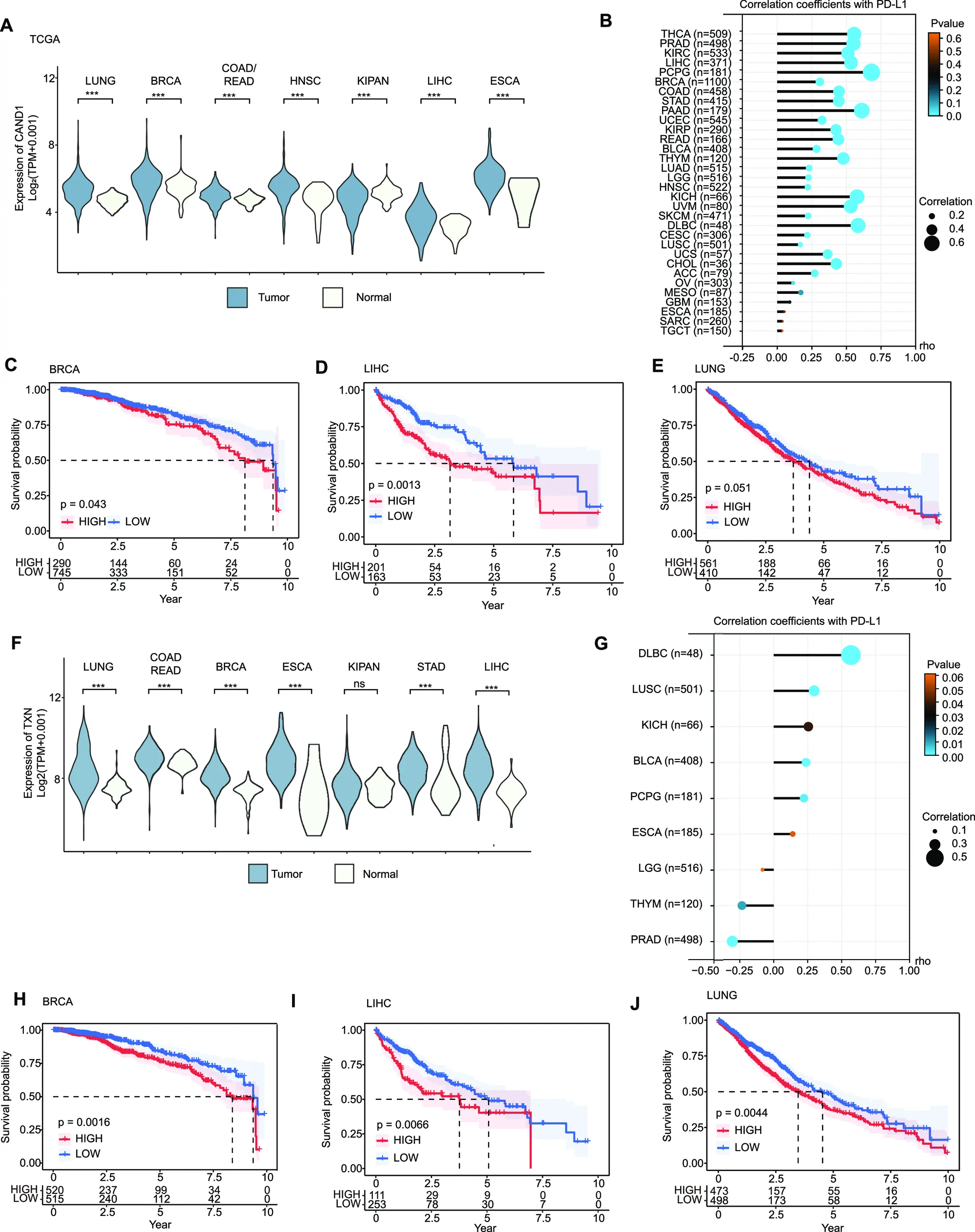
Validation of PD-L1-interacting protein expression differences and survival prognosis analysis using TCGA data. (A, F) Differential expression of CAND1 (A) and TXN (F) in tumor and adjacent nontumor tissues based on The Cancer Genome Atlas (TCGA) database. Statistical significance was denoted as *P < 0.05, **P < 0.01, and ***P < 0.001. (B, G) Correlations between CAND1 (B) or TXN (G) and PD-L1 expression across different tumor types, analyzed using the TIMER database based on TCGA data. (C–E) Kaplan–Meier analyses showing the association between CAND1 mRNA expression and overall survival in breast invasive carcinoma (BRCA) (C), liver hepatocellular carcinoma (LIHC) (D), and lung cancer (LUNG) (E). (H–J) Kaplan–Meier analyses showing the association between TXN mRNA expression and overall survival in BRCA (H), LIHC (I), and LUNG (J).

### Positive Correlation Between CAND1, TXN, FBXO2 and PD-L1 Expression

CAND1 is a core component of the SCF (SKP1-Cullin-F-box) ubiquitin ligase complex, which facilitates the dynamic exchange of F-box proteins within the SCF complex to regulate substrate specificity and protein degradation (Figure 6A). In our module-based proteomic analysis, six F-box (FBX) family proteins significantly associated with PD-L1 were identified in modules 0 and 3, including FBXL4, FBXW9, FBXL15, FBXO2, FBXO21, FBXO22, and FBXO38 (Figure 6B). Among these candidates, FBXO22 and FBXO38 have previously been reported to be closely linked to PD-L1 regulation. Based on these findings, we hypothesized that FBXO2 may also participate in CAND1-mediated PD-L1 regulation. IHC analysis revealed a significant positive correlation between FBXO2 and CAND1 protein levels in tumor tissues (Figure 6C).

**Figure 6:**
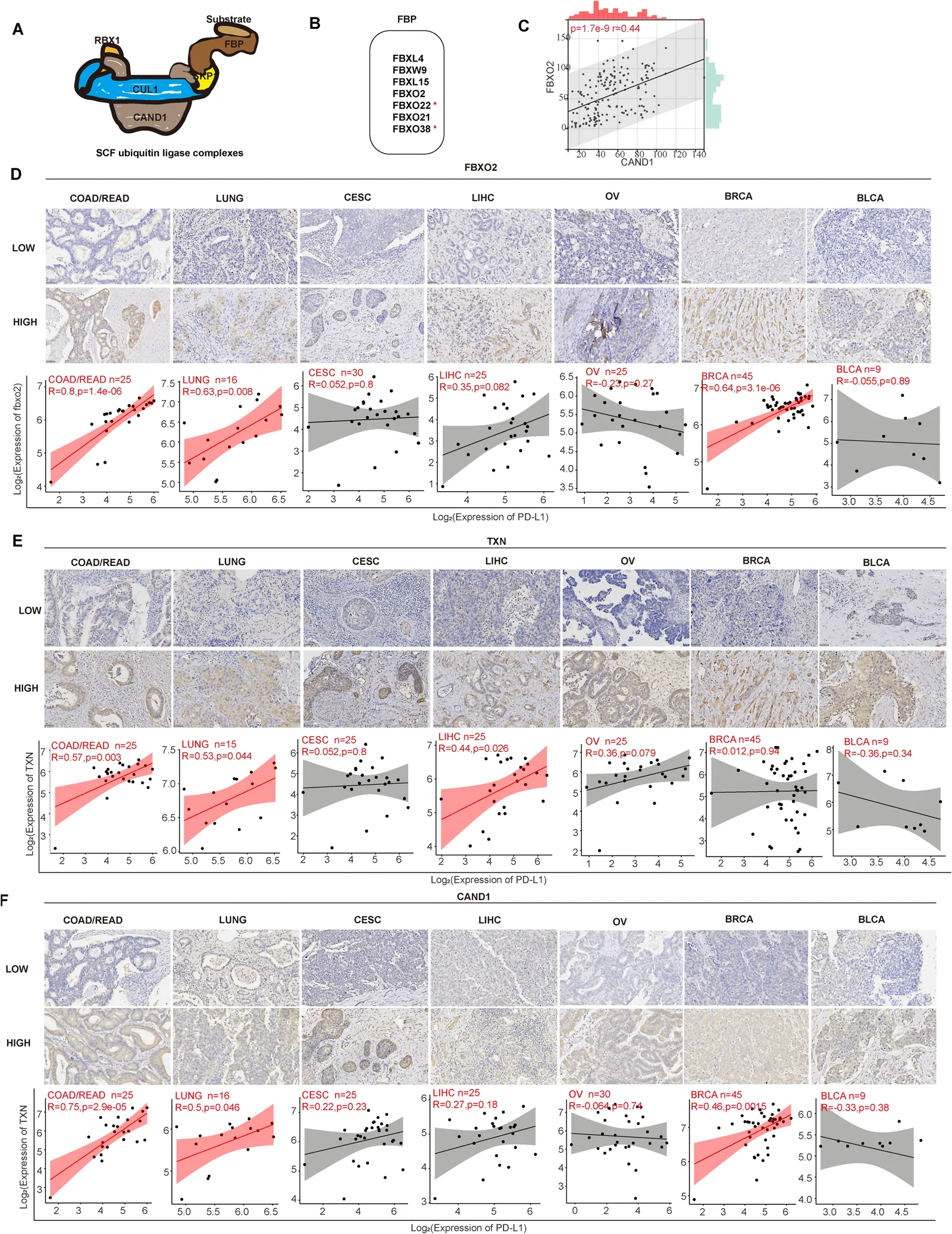
Immunohistochemical analysis of the correlation between PD-L1 and its interacting proteins. (A) Schematic illustration of the SCF (SKP1-Cullin-F-box) ubiquitin ligase complex, highlighting the role of CAND1 in regulating F-box protein exchange and substrate recognition. (B) F-box (FBX) family proteins identified from pan-cancer proteomic analysis and PD-L1-associated modules, including FBXL4, FBXW9, FBXL15, FBXO2, FBXO21, FBXO22, and FBXO38. * Indicates proteins previously reported to regulate PD-L1 expression. (C) Correlation analysis between CAND1 and FBXO2 protein expression levels evaluated by IHC in tumor tissues (magnification: 20×). (D–F) Representative IHC images of FBXO2 (D), TXN (E), and CAND1 (F). The correlations between PD-L1 expression and CAND1/FBXO2/TXN expression were evaluated.

We further examined the relationships among FBXO2, CAND1, TXN, and PD-L1 expression using IHC across multiple cancer types (Figure 6D–F). FBXO2 expression was significantly positively correlated with PD-L1 in colon cancer, lung cancer, and breast cancer (Figure 6D). TXN expression showed a positive correlation with PD-L1 levels in colon cancer, lung cancer, and liver hepatocellular carcinoma (Figure 6E). Expression of CAND1 was significantly correlated with PD-L1 protein expression in colorectal cancer, lung cancer and breast cancer (Figure 6F).

### Determination of CAND1, TXN, FBXO2 and the Immunosuppressive Microenvironment

We next determined the association of expressions of CAND1, TXN, FBXO2 and immune cell infiltration in human cancers. Immune checkpoint markers including PD1, CTLA4, TIGIT, and TIM3, as well as markers for the common immune cell populations including T cells (CD3, CD4, CD8, FoxP3, and Granenzyme B), B cells (CD19 and CD138), macrophages (CD68, CD163, and CD86), neutrophils (CD15), hematopoietic

stem cells (CD34), and NK cells (CD56), were tested by IHC. Correlation analyses revealed distinct immune infiltration patterns associated with high expression of TXN, FBXO2, and CAND1 (Figure 7A, Supporting Table 9). Interestingly, tissues with high TXN expression showed increased infiltration of CD15+, GrB+, and TIM3+ cells (Figure 7A–B). In tissues with high FBXO2 expression, immune markers significantly correlated with CD19, CD68, CTLA4, and FOXP3. In tissues with high CAND1 expression, CD8, CD15, CD19, and FOXP3 expression was elevated, while the immune checkpoint marker TIGIT was relatively downregulated (Figure 7C–D). These data suggest that tumors with high TXN, FBXO2, and CAND1 expression tend to exhibit inflammatory and immunosuppressive characteristics.

**Figure 7:**
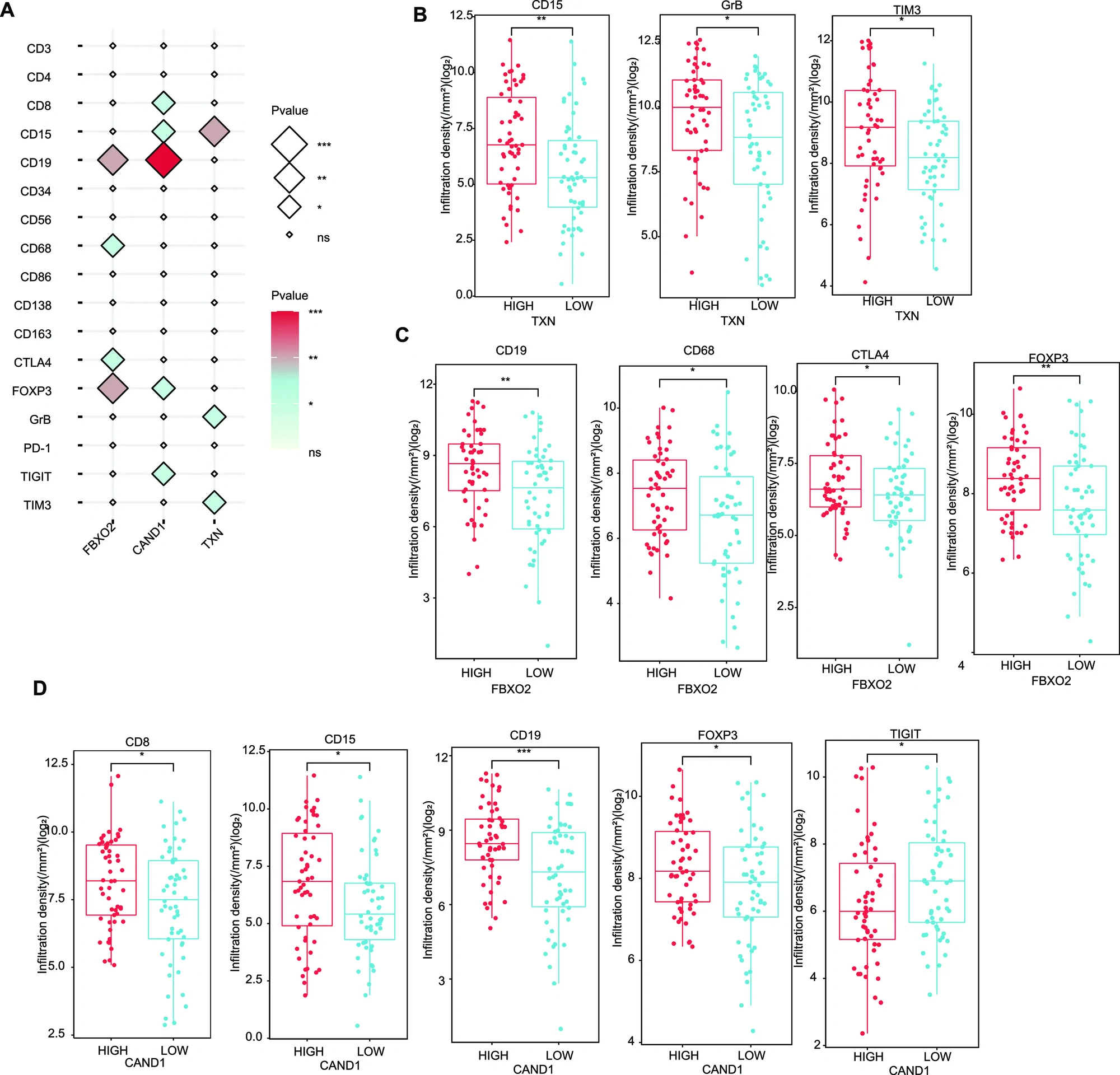
Correlation analysis of PD-L1-interacting protein expression with immune cell infiltration and immune checkpoint molecules. (A) Overview of the correlation landscape between the expression of PD-L1-interacting proteins (CAND1, FBXO2, and TXN) and immune-related markers, including immune cell infiltration markers and immune checkpoint molecules. Dot size and color indicate the strength and direction of correlations, respectively, with statistical significance denoted accordingly. (B) Comparison of immune cell infiltration levels between tumors with high and low TXN expression, showing significant differences in CD15+, GrB+ cytotoxic cell, and TIM3 immune checkpoint expression. (C) Comparison of immune marker infiltration between tumors with high and low FBXO2 expression, demonstrating significant associations with CD19, CD68, CTLA4, and FOXP3 expression. (D) Comparison of immune marker infiltration between tumors with high and low CAND1 expression, showing increased infiltration of CD8+, CD15+, CD19+, and FOXP3+ cells, accompanied by reduced TIGIT expression (analyzed by independent samples *t* test).

### Regulatory Effect of CAND1, TXN and FBXO2 on PD-L1 Protein Expression

We next validated the interactions between CAND1, TXN, FBXO2 and PD-L1, as well as their regulatory roles in modulating PD-L1 expression. CoIP data showed that Cand1 was able to bind to PD-L1 in 293T and KYSE-150 cells (Figure 8A–B). The knockdown of CAND1 resulted in the downregulation of PD-L1 protein in 786-O cells (Figure 8C–D). The interaction between TXN and PD-L1 was confirmed in 786-O cells (Figure 8E). The expression of PD-L1 protein was markedly decreased in 786-O and KYSE-150 cells treated with TXN siRNAs (Figure 8F–I). In KYSE-150 cells, the silence of FBXO2 resulted in the reduction of PD-L1 protein, whereas the overexpression of FBXO2 induced the PD-L1 protein expression (Figure 8J–K).

**Figure 8:**
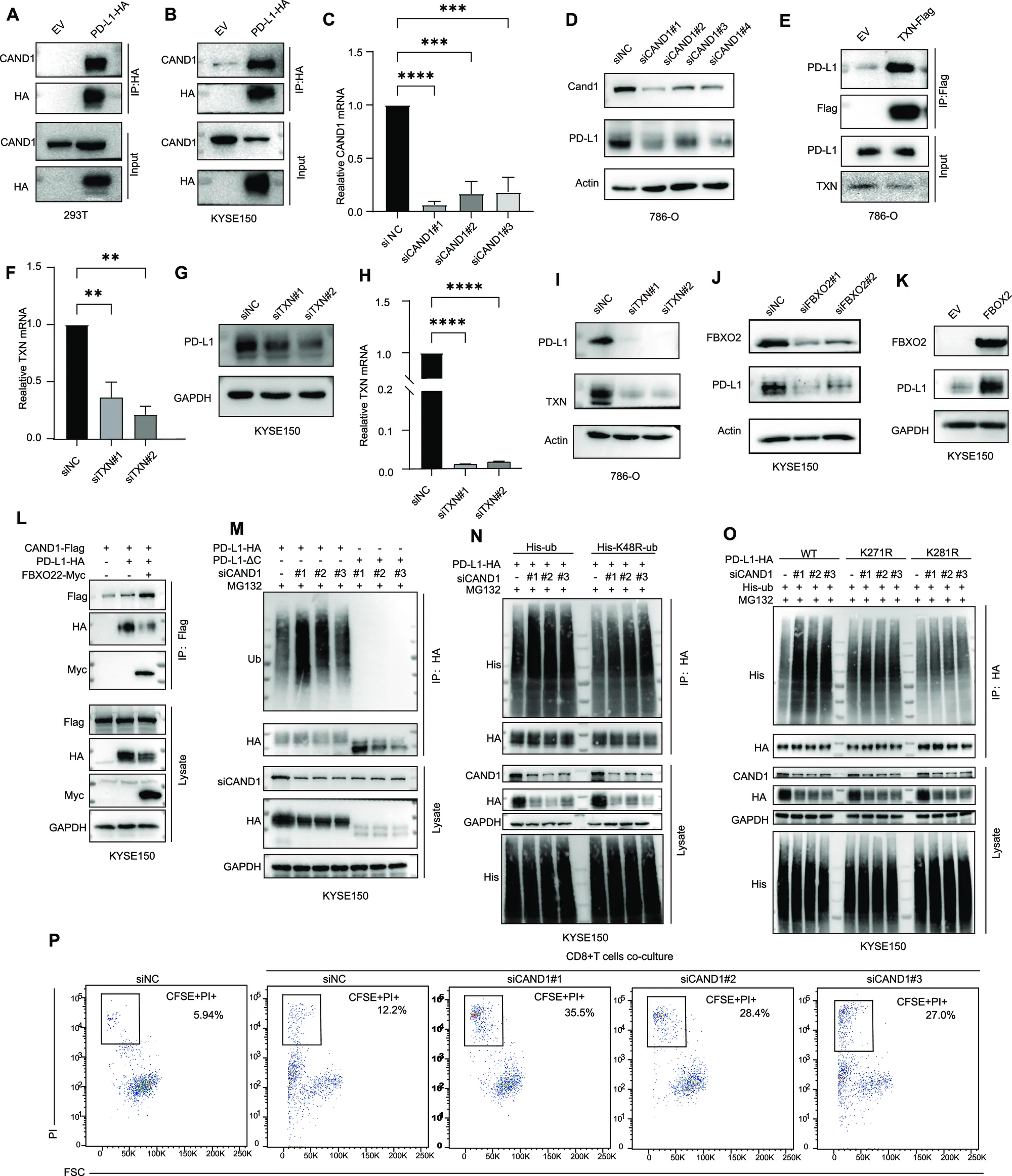
Verification of the regulatory association between PD-L1 and its interacting proteins. (A–B) Co-immunoprecipitation assays demonstrating a physical interaction between CAND1 and PD-L1 in 293T (A) and KYSE-150 (B) cells. (C–D) CAND1 knockdown reduced PD-L1 protein levels in 786-O cells. (E) Co-IP analysis showing that TXN directly interact with PD-L1 in 786-O cells. (F–I) TXN knockdown reduced PD-L1 protein levels in KYSE-150 (F, G) and 786-O (H, I) cells. (J) FBXO2 knockdown reduced PD-L1 protein levels in KYSE-150. (K)­FBXO2 overexpression increased PD-L1 protein levels. (L) Cells were cotransfected with CAND1-Flag, PD-L1-HA and FBXO22-Myc constructs. Cell lysates were immunoprecipitated using anti-Flag antibody. (M) Cells expressing PD-L1-HA or intracellular domain-truncated PD-L1-ΔC along with His-ubiquitin were transfected with control siRNA or siCAND1. Following MG132 treatment, ubiquitinated proteins were enriched and analyzed by immunoblotting. (N) KYSE150 cells were transfected with PD-L1-HA plus wild-type His-ubiquitin or His-K48R-ubiquitin, followed by CAND1 knockdown and MG132 incubation. (O) KYSE150 cells were transfected with His-ubiquitin and HA-tagged wild-type PD-L1, PD-L1-K271R or PD-L1-K281R mutants, together with siCAND1, and treated with MG132. (P) CAND1 knockdown enhances CD8+ T cell-mediated tumor cell killing. Representative flow cytometry plots showing PI-positive tumor cells within the CFSE-positive tumor cell population after coculture with CD8+ T cells. Tumor cells were labeled with CFSE, followed by coculture with CD8+ T cells. Cell death induced by CD8+ T cell-mediated killing was assessed by quantifying the percentage of CFSE+PI+ cells. The percentages indicate CFSE+PI+ tumor cells in each group.

Considering that CAND1 governs the assembly and dynamic exchange of SCF complexes, we hypothesized that CAND1 modulates PD-L1 stability via an SCF-dependent ubiquitination mechanism. Co-immunoprecipitation assays confirmed that CAND1, PD-L1, and FBXO22 (an F-box subunit of the SCF ubiquitin ligase complex) coexist in the same protein complex, indicating a functional interplay among these proteins (Figure 8L). CAND1 knockdown markedly elevated PD-L1 ubiquitination. Truncation of the PD-L1 intracellular domain substantially diminished its ubiquitination, verifying that the cytoplasmic region of PD-L1 is indispensable for this ubiquitin-dependent regulatory mechanism (Figure 8M). We next characterized the ubiquitin linkage type underlying CAND1-mediated PD-L1 regulation. Using ubiquitin mutant constructs, we found that CAND1 depletion specifically enhances K48-linked ubiquitination of PD-L1 (Figure 8N). In addition, mutagenesis of the PD-L1 intracellular lysine residues K271 and K281 markedly attenuated PD-L1 ubiquitination, identifying these two residues as key ubiquitination sites responsible for CAND1-dependent PD-L1 regulation (Figure 8O). Functionally, T cell-mediated tumor killing assays revealed that CAND1 knockdown significantly potentiates T cell cytotoxicity against tumor cells (Figure 8P–Q).

## Discussion

The blockade of the PD-L1/PD-1 signaling, which mitigates immune evasion, has greatly advanced the development of cancer immunotherapy. Building on the remarkable success of clinical trials, ten PD-1 antibodies and three PD-L1 antibodies have been approved for the treatment of various cancers.^17^ However, the low response rate to ICB therapy remains a major challenge. For most cancer patients, the PD-1/PD-L1 pathway is not the limiting factor in antitumor immunity, and blocking the axis alone is insufficient to elicit a robust antitumor immune response.^18^ Accumulating evidence indicates that chemotherapy, radiotherapy, and other immunomodulators can synergize with anti-PD-1/PD-L1 therapies by enhancing cancer antigen release and effector cell activity.^9,19–27^ Elucidating the regulatory network of PD-L1 has attracted considerable attention. In this study, we integrated pancancer proteomics, proximity proteomics and immunohistochemistry to map the protein–protein interaction (PPI) network governing PD-L1 expression.

Proper subcellular localization is critical for PD-L1 to exert its biological functions. Our findings highlight a distinct functional division between the N- and C-terminal domains of PD-L1 in coordinating its trafficking, stability, and functional regulation. The N-terminal domain of PD-L1 contains a signal peptide sequence that is essential for targeting the protein to specific cellular compartments, thereby ensuring correct membrane localization and functional competence. In this study, we identified potential regulatory molecules of PD-L1 and confirmed that CAND1, FBXO2, and TXN were involved in the PD-L1 regulatory network. Immunohistochemical analyses revealed a strong correlation between the expression of these proteins and PD-L1 in tumor tissues, particularly in breast cancer, liver cancer, and lung cancer. However, due to tumor heterogeneity, varying correlations between CAND1 and PD-L1 expression among different malignancies.^9^ We found that CAND1 bound to PD-L1 and contributes to the stabilization of PD-L1 in cancer cells. CAND1 is a key assembly factor of the SCF (SKP1-CUL1-F-box protein) E3 ubiquitin ligase complex.^9^ The exchange activity of CAND1 is coupled to the neddylation cycle.^28,29^ SCF ubiquitin ligases control diverse cellular and organismal processes, and their dysregulation is associated with numerous pathologies.^30^

Dysregulation of F-box protein-mediated proteolysis has been implicated in the development and progression of various human malignancies. Notably, small-molecule inhibitors targeting F-box proteins have demonstrated promising therapeutic potential in preclinical and early clinical studies.^31^ Studies have shown that a series of FBX family proteins, such as FBXO6, FBXO22, and FBXO38, are involved in regulating PD-L1 stability.^32–34^ As a member of the F-box family, FBXO2 plays a significant role in tumor development and progression; it has been shown to promote cancer progression in glioma and ovarian cancer.^35,36^ However, FBXO2 has not been previously reported to be involved in PD-L1 expression regulation. We speculate that FBXO2 may influence PD-L1 expression by participating in the SCF ubiquitin ligase complex. Additionally, since both CAND1 and FBXO2 are components of the SCF ubiquitin complex, we hypothesize that they may act synergistically to coregulate PD-L1 levels.

As an F-box protein, FBXO22 acts as the substrate-recognition subunit of SCF E3 ubiquitin ligase complexes and has been shown to drive ubiquitin-dependent PD-L1 degradation.^33^ Multiple prior investigations, including work demonstrating that ARIH1 signaling reinforces antitumor immunity by targeting PD-L1 for proteasomal breakdown, have established that PD-L1 undergoes K48-linked ubiquitination at defined lysine residues located within its intracellular domain.^37^ In line with these observations, our co-IP results revealed that CAND1, PD-L1, and FBXO22 reside within a shared protein complex. We found that CAND1 depletion augments K48-linked PD-L1 ubiquitination, while truncation of the PD-L1 intracellular domain substantially blunts this modification. Moreover, mutagenesis of K271 or K281 impaired PD-L1 ubiquitination, implicating these lysine residues in CAND1-dependent control of PD-L1 stability. Collectively, these data support a working model whereby CAND1 tunes PD-L1 abundance by modulating the activity of SCF-F-box ubiquitin ligase machineries, with FBXO22 acting as a candidate mediator governing PD-L1 ubiquitination.

Our results further showed that elevated expression of CAND1, FBXO2, or TXN was associated with increased expression of immune checkpoint molecules, including CTLA4, TIGIT, and TIM3, as well as higher infiltration of immunosuppressive immune cells, such as regulatory T cells, B cells, and macrophages. These findings suggest that CAND1, FBXO2, and TXN may participate in shaping an immunosuppressive tumor microenvironment, at least in part through their regulation of PD-L1 expression and stability. Together, our study defines the proximal proteomic landscape of PD-L1 and identifies CAND1, FBXO2, and TXN as clinically relevant PD-L1-associated regulators. In particular, CAND1 appears to stabilize PD-L1 by limiting K48-linked ubiquitination and proteasomal degradation, thereby contributing to tumor immune evasion. Targeting the CAND1/FBXO22/PD-L1 regulatory axis may therefore provide a potential strategy to remodel the tumor immune microenvironment and improve the efficacy of PD-1/PD-L1-based immunotherapy.

## Supplementary Material





## Data Availability

The mass spectrometry proteomics data have been deposited in the PRIDE Archive (https://www.ebi.ac.uk/pride/archive/) via the PRIDE partner repository with the data set identifier PXD077446.

## Notes

Supporting Figure 1: PD-L1 proximal proteome identified by PhastID in HepG2 cells. Supporting Figure 2: PD-L1-associated trend clustering and functional enrichment analysis in an external ESCC cohort (PDF)
Supporting Tables 1–4: Sample group information and protein expression matrix identified by PhastID. Supporting Table 5: Proteomic data of pan-cancer samples. Supporting Table 6: Quantitative results of PD-L1 immunohistochemistry in pan-cancer samples. Supporting Table 7: Proteomic data of ESCC samples. Supporting Table 8: Quantitative results of PD-L1 immunohistochemistry in 82 ESCC samples. Supporting Table 9: Quantitative results of immunohistochemistry for immune checkpoint-related molecules (XLSX)
