# Supplementary material for: Proximal Proteomics Reveals Potential Regulators of Programmed Death-Ligand 1 in Human Cancer
Source: ACS Omega. 2026 Aug 13;11(33):50389–405. doi: 10.1021/acsomega.6c06622 (PMC13520555; doi:10.1021/acsomega.6c06622)
Supplement: Supplementary file 1 [file ao6c06622_si_001.pdf]

Proximal Proteomics Reveals Potential Regulators of Programmed Death-Ligand 1 in human cancer

Fujin Shi[1,2,#], Danya Liu[2,#], Yaxiong Liu[3,#] , Fan Li[1], Chris Zhiyi Zhang[2], Qiuli Li[4], Qiu-Hong Tian[1,*]

[1]Department of Medical Oncology, The First Affiliated Hospital, Jiangxi Medical College, Nanchang University, China.

[2]MOE Key Laboratory of Tumor Molecular Biology and State Key Laboratory of Bioactive Molecules and Druggability Assessment, Institute of Life and Health Engineering, College of Life Science and Technology, Jinan University, Guangzhou, China.

[3]Breast Disease Center, The First Affiliated Hospital, Jiangxi Medical College, Nanchang University, China.

[4]Department of Head and Neck, Sun Yat-sen University Cancer Center, Guangzhou 510060, China

[#]These authors contributed equally to this work.

*Correspondence: Qiu-Hong Tian, Department of Medical Oncology, First Affiliated Hospital of Nanchang University, Nanchang, China, Email: ndyfy02125@ncu.edu.cn

Funding: This work was supported by The National Natural Science Foundation of China (No. 82273109) and the Natural Science Foundation of Jiangxi Province (20232BAB206100).

Author contributions: Tian QH designed the study and prepared the manuscript. Shi F, Liu D, Liu Y and Li F conducted the experiments and analyzed the data. Shi F, Zhang CZ, and Tian QH wrote and edited the manuscript. Tian QH conceived and supervised the research.

Competing interests: The authors declare that they have no competing interests.

Figure S1. PD-L1 proximal proteome identified by PhastID in HepG2 cells

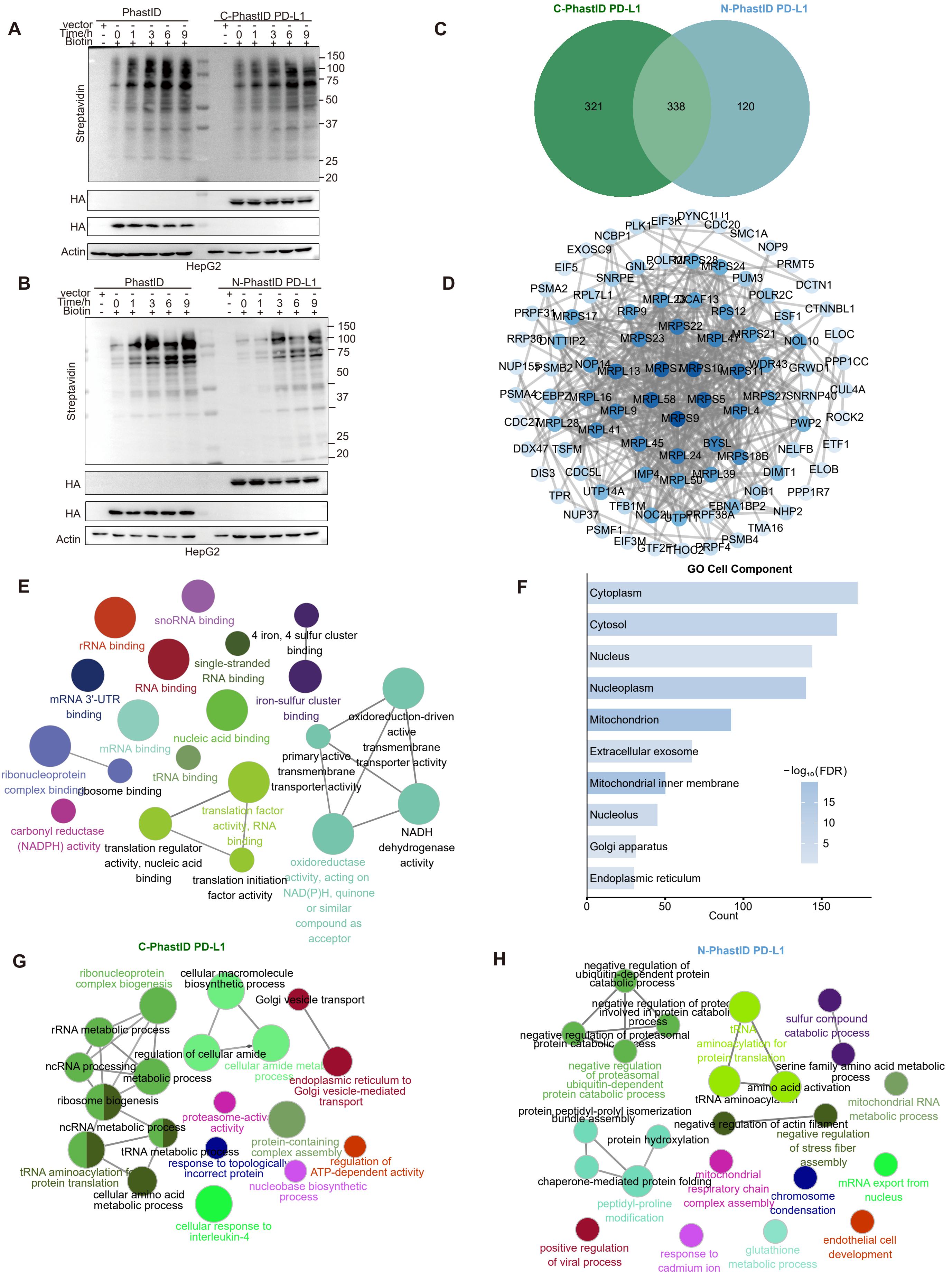

Figure S1. PD-L1 proximal proteome captured by PhastID proximity labeling in HepG2 cells. (A-B) Validation of biotin labeling mediated by C-PhastID-PD-L1 and N-PhastID-PD-L1 in HepG2 cells. Cells expressing PD-L1 with C- or N-terminal PhastID tags were incubated with biotin for the indicated durations, and biotinylated proteins were detected via streptavidin immunoblotting. (C) Venn diagram illustrating the overlap of proteins identified by C-PhastID-PD-L1 and N-PhastID-PD-L1 labeling in HepG2 cells. In total, 120 proteins were common to both datasets, while 321 and 338 proteins were uniquely identified in the C-terminal and N-terminal PhastID-PD-L1 datasets, respectively. (D) Protein-protein interaction network of shared PD-L1 proximal proteins recovered from both C- and N-PhastID-PD-L1 labeling. The network was built using the STRING database, retaining only high-confidence interactions. (E-F) GO molecular function and cellular component enrichment analyses of the overlapping proteins. (G-H) GO biological process enrichment analyses for proteins specific to C-PhastID-PD-L1 and N-PhastID-PD-L1, respectively.

Figure S2 PD-L1-associated trend clustering analysis in an external ESCC cohort

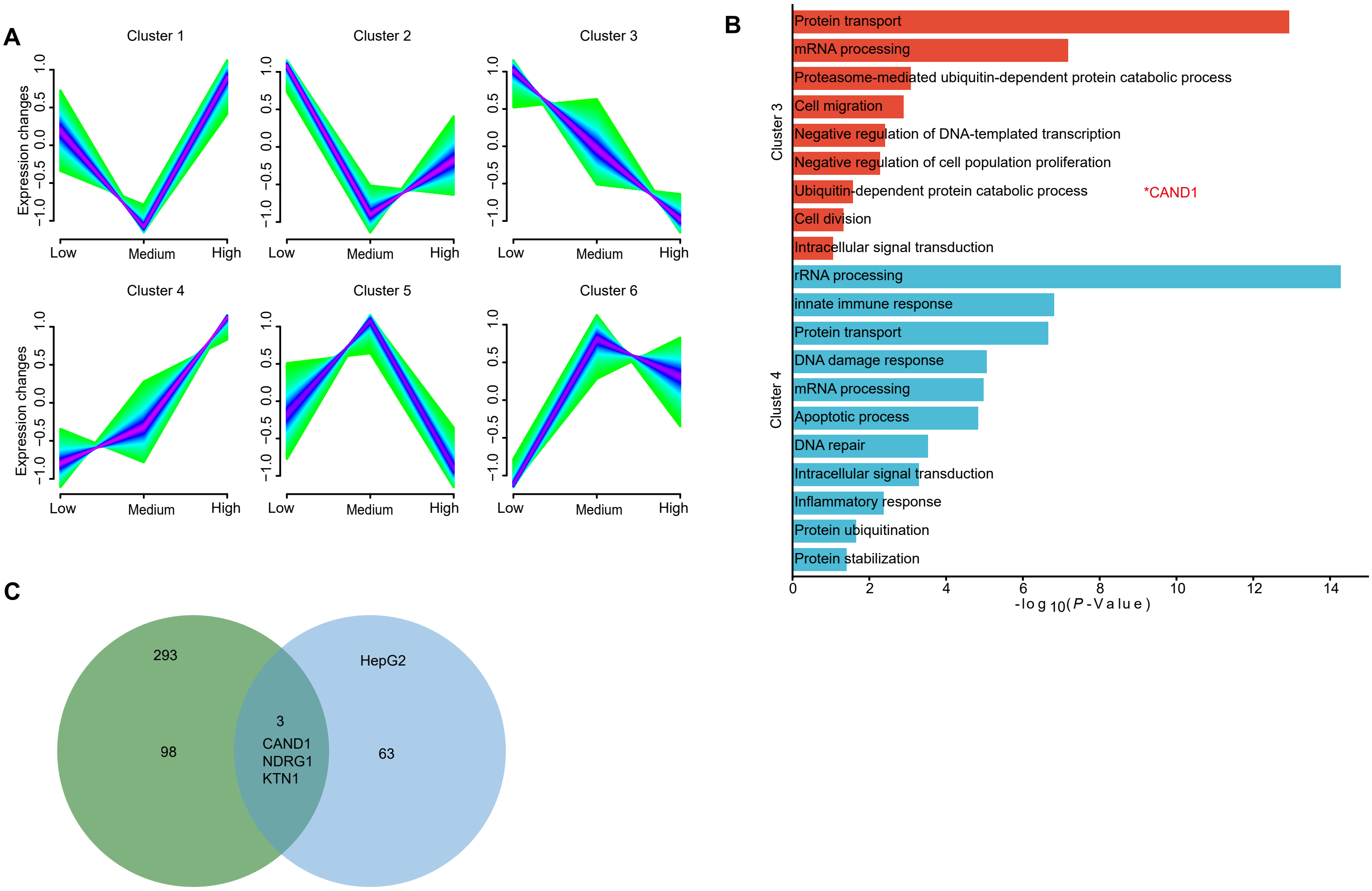

Figure S2. PD-L1-associated trend clustering and functional enrichment analysis in an external ESCC cohort. (A) Trend clustering analysis of proteins quantified in an external ESCC proteomic dataset according to PD-L1 expression levels. Proteins were grouped into six clusters based on their expression patterns across PD-L1-low, PD-L1-medium, and PD-L1-high groups. (B) GO biological process enrichment analysis of proteins in clusters3 and clusters4. GO biological process terms enriched in the CAND1-containing cluster included proteasome-mediated ubiquitin-dependent protein catabolic process, protein ubiquitination, indicating that CAND1 is associated with PD-L1-related protein homeostasis and ubiquitin-proteasome pathways in ESCC.(C)Venn diagram showing the overlap of PD-L1-associated candidate proteins identified from the 293T- and HepG2-based integrated analyses.
